# Serial ctDNA Analysis for Monitoring Molecular Response and Acquired Resistance in Metastatic NSCLC and HR+/HER2‐Negative Breast Cancer: A Real‐World Study

**DOI:** 10.1002/cam4.72208

**Published:** 2026-10-01

**Authors:** Franciele Hinterholz Knebel, Maurício Fernando Silva Almeida Ribeiro, Rudinei Diogo Marques Linck, Isabela Flauzino Ferreira, Cibele Masotti, Rodrigo Saddi, Karina Perez Sacardo, Felipe Sales Nogueira Amorim Canedo, Leandro Jonata Carvalho Oliveira, Daniele Coelho Duarte, Paula Fontes Asprino, Ernande Xavier dos Santos, Elisângela Monteiro Coser, Dimitrios Kleftogiannis, Louise J. Barber, Marco Gerlinger, João Victor Machado Alessi, Andrea Kazumi Shimada, Fernando Costa Santini, Ciro Eduardo de Souza, Olavo Feher, Gilberto de Castro Junior, Frederico Perego Costa, Max Senna Mano, Artur Katz, Anamaria Aranha Camargo, Fabiana Bettoni

**Affiliations:** ^1^ Molecular Oncology Center Hospital Sírio‐Libanês São Paulo Brazil; ^2^ Oncology Center Hospital Sírio‐Libanês São Paulo Brazil; ^3^ Bioinformatics Team, Centre for Evolution and Cancer The Institute of Cancer Research London UK; ^4^ Translational Oncogenomics Lab The Institute of Cancer Research London UK

**Keywords:** acquired resistance, circulating tumor DNA, hormone receptor‐positive breast cancer, non‐small cell lung cancer

## Abstract

Circulating tumor DNA (ctDNA) is a promising biomarker for disease monitoring, yet evidence of its clinical utility in routine care remains limited. We evaluated the utility of serial ctDNA monitoring using digital PCR (ddPCR) for detecting acquired resistance (AR) and monitoring response in metastatic non‐small cell lung cancer (NSCLC) and breast cancer (MBC). This real‐world, longitudinal study included 48 metastatic patients (42 with *EGFR*‐mutant NSCLC; 6 with HR+/HER2‐negative MBC) treated at a Brazilian oncology reference center. Serial plasma samples were analyzed by ddPCR for activating and resistance mutations at specific time points. In the NSCLC cohort, *EGFR* T790M was detected in 48.4% of patients who progressed on 1st/2nd‐generation TKIs. For patients on 3rd‐generation TKIs, *EGFR* C797S was the main AR mechanism (47%), followed by *PIK3CA*, *BRAF* mutations, and *ERBB2*/*EGFR* amplifications. Longitudinal analysis anticipated radiological progression by approximately 5 months. Early molecular response, characterized by mutation clearance, preceded radiological response. In the MBC cohort, hotspot *ESR1* mutations (D538G, L536R, Y537S) were identified in 50% of patients. Variations in ctDNA fractional abundance predicted disease progression up to 9 months before radiological confirmation. This study provides real‐world evidence that ddPCR‐based ctDNA monitoring enables early detection of resistance and predicts disease progression significantly earlier than standard imaging in both NSCLC and MBC. Despite the limited sample size, particularly in the MBC cohort, these real‐world findings support liquid biopsy as a minimally invasive tool with the potential to guide therapeutic decisions and inform personalized management in precision oncology. Larger prospective studies are needed to confirm these observations.

**Abbreviations**
AI: aromatase inhibitor
AR: acquired resistance
ASCO: American Society of Clinical Oncology
CDK4/6: cyclin‐dependent kinase 4/6
cfDNA: circulating cell‐free DNA
ctDNA: circulating tumor DNA
ddPCR: droplet digital PCR
DP: disease progression
EGFR: epidermal growth factor receptor
EGFR‐act mutation: EGFR‐activating mutation
ER: estrogen receptor
ET: endocrine therapy
FA: fractional abundance
HR+: hormone receptor‐positive
IASLC: International Association for the Study of Lung Cancer
MBC: metastatic breast cancer
NGS: next‐generation sequencing
NSCLC: non‐small cell lung cancer
OS: overall survival
PFS: progression‐free survival
RNAseP: Ribonuclease P
SCLC: small cell lung cancer
SERDs: selective estrogen receptor degraders
TKI: tyrosine kinase inhibitor

## 1 Introduction

The detection of circulating tumor DNA (ctDNA) in plasma has become a promising biomarker for initial tumor genotyping, monitoring molecular disease response, and predicting and characterizing acquired resistance (AR) in the era of precision oncology [1, 2, 3]. While several studies have confirmed the clinical validity of ctDNA monitoring, real‐world studies are needed to complement clinical trial data by validating clinical utility across diverse patient populations and the complexities of routine care [3, 4, 5, 6, 7, 8, 9, 10]. Although tissue biopsy remains the gold standard for tumor molecular analysis, it is associated with clinical complications, sampling difficulties, and limited capture of tumor heterogeneity. Liquid biopsy, by contrast, is minimally invasive and captures spatial and temporal heterogeneity with high sensitivity [1, 2]. Compared with next‐generation sequencing, digital PCR offers a faster, lower‐cost approach for monitoring well‐characterized, recurrent alterations, such as those driving resistance in lung and breast cancer.

In non‐small cell lung cancer (NSCLC), the epidermal growth factor receptor (*EGFR*) is one of the most common driver genes; its prevalence varies markedly by ethnicity and geographic region, occurring in approximately 47% of NSCLC adenocarcinomas in Asian populations, compared with approximately 10%–15% in Western/Caucasian populations [11, 12]. Patients with *EGFR*‐driven NSCLC are eligible for targeted therapies that have revolutionized the treatment of advanced *EGFR*‐mutated NSCLC [13, 14, 15, 16]. Several clinical trials have shown improved progression‐free survival (PFS) and/or overall survival (OS) with 1st‐generation (gefitinib and erlotinib), 2nd‐generation (afatinib), and 3rd‐generation (osimertinib) EGFR TKIs (Tyrosine kinase inhibitors) compared to chemotherapy [14, 17, 18, 19, 20]. However, tumors inevitably develop resistance to TKI therapies, driven by secondary mutations in the *EGFR* gene in nearly 50% of cases [21]. Additional resistance mechanisms to 1st‐ and 2nd‐generation TKIs are also present at a lower frequency, including *MET* and *ERBB2* amplification, *PIK3CA* mutations, *BRAF* V600E, and histologic transformation from NSCLC to SCLC (small cell lung cancer) [22, 23, 24]. The heterogeneity of resistance mechanisms to osimertinib includes *EGFR* and *MET* amplification, *EGFR* C797S mutation, off‐target mutations in *PIK3CA*, *KRAS*, and *ERBB2*, *ROS1* fusions, as well as histologic transformation [25, 26].

Molecular assessment of the tumor is crucial at disease progression to guide personalized treatment strategies in NSCLC patients. The increasing number of NSCLC oncogene alterations identified and targeted therapies developed have enabled ctDNA analysis as a complementary tool to tissue genotyping in treatment decisions.

The International Association for the Study of Lung Cancer (IASLC) updated its statement on liquid biopsy in 2021 [27]. For treatment‐naive patients with advanced or metastatic NSCLC, the IASLC recommends using liquid biopsy alongside tissue biopsy when, even if a tissue sample is available for initial tumor genotyping, the analysis is incomplete, the sample is inadequate, or scarce. If no tissue sample is available for genotyping, the IASLC advises a “plasma first” approach. However, in cases of disease progression (DP) after targeted therapy, the IASLC suggests considering a “plasma first” approach to evaluate AR mechanisms. If the analysis provides uninformative results, a tissue rebiopsy and analysis are then recommended.

For patients with hormone receptor‐positive (HR+), human epidermal growth factor receptor 2 (HER2)‐negative locally advanced or metastatic breast cancer (MBC), cyclin‐dependent kinase 4/6 (CDK4/6) inhibitors combined with endocrine therapy (ET), either aromatase inhibitors (AI) or fulvestrant, are the recommended first‐line standard treatment [28, 29], although resistance is nearly universal and multiple post‐CDK4/6i treatment strategies are under active investigation [30]. Activating mutations in *ESR1*, which encodes the estrogen receptor alpha protein, are a major mechanism of acquired resistance to ET, affecting 20%–40% of MBC patients [31], and have led to the development of oral selective estrogen receptor degraders (SERDs) such as elacestrant and camizestrant [32, 33]. Based on the EMERALD trial, in which *ESR1* mutation status was assessed by ctDNA, the 2023 ASCO guideline recommends *ESR1* mutation testing—preferably by ctDNA—to guide therapy at recurrence or progression on ET [34], and serial ctDNA monitoring for emergent *ESR1* mutations, as demonstrated in the PADA‐1 trial, may further guide treatment switching [35]. Most recently, the phase 3 SERENA‐6 trial demonstrated that switching to camizestrant upon ctDNA detection of an emerging *ESR1* mutation—before clinical or radiological progression—while continuing CDK4/6i, improved progression‐free survival in the first‐line setting [36], directly supporting the rationale for serial ctDNA monitoring explored in the present study.

Here, we conducted a real‐world study to evaluate the clinical utility of serial ctDNA monitoring by droplet digital PCR (ddPCR) for early detection of acquired resistance and molecular treatment response, and to characterize the genetic alterations associated with therapy resistance in patients with metastatic NSCLC and HR+, HER2‐negative MBC treated at Hospital Sírio‐Libanês, a leading private oncology referral center in Brazil.

## 2 Methods

### 2.1 Patients

Patients were enrolled from all individuals with metastatic EGFR‐mutant NSCLC starting EGFR‐TKI therapy (NSCLC Cohort), or HR+/HER2‐negative MBC starting ET ± iCDK4/6 (MBC Cohort), at our institution who provided written informed consent to serial research blood collection under an IRB‐approved translational research study (HSL2015‐22). ctDNA monitoring was investigational and did not guide real‐time clinical decision‐making, allowing an unbiased retrospective assessment of its predictive value against the standard‐of‐care radiological follow‐up. Inclusion criteria: Patients aged ≥ 18 years with histologically confirmed metastatic NSCLC harboring a documented EGFR‐activating mutation who were initiating treatment with an EGFR‐TKI, or with histologically confirmed HR+/HER2‐negative metastatic breast cancer initiating ET alone or with an iCDK4/6; written informed consent for serial blood collection and genomic analysis. Exclusion criteria: failure to consent to study participation. All NSCLC patients had a histologically confirmed diagnosis of lung adenocarcinoma. Baseline EGFR‐activating mutations, determined by tissue genotyping at diagnosis, included EGFR exon 19 deletion, EGFR exon 18 mutations, EGFR exon 20 mutations, and EGFR exon 21 mutations, and were the basis for eligibility for first‐line or subsequent EGFR‐TKI therapy. Serial blood samples from both cohorts were drawn throughout the treatment period from December 2016 to March 2021, and for most patients, samples were collected monthly. Radiologic progressive disease was defined according to RECIST v1.1 criteria [37].

### 2.2 Plasma Processing and Circulating Cell‐Free DNA Extraction

Peripheral blood samples (20 mL) were collected into tubes containing EDTA, and plasma was separated from blood within 2 h as previously described [38]. Circulating cell‐free DNA (cfDNA) was isolated using the QIAamp MinElute Virus Vacuum Kit (Qiagen, Hilden, Germany) and stored at −80°C until needed.

### 2.3 Droplet Digital PCR (ddPCR)

cfDNA was quantified by ddPCR using the *RNAseP* (Ribonuclease P) Copy Number Reference Assay (Thermo Fisher Scientific, Carlsbad, CA, USA). A total of 3000 genome equivalents (~10 ng of cfDNA) and 1500 genome equivalents (~5 ng of cfDNA) were analyzed for plasma samples from lung and breast cancer patients, respectively, to achieve a detection sensitivity of 0.2%. Probes and primers for *EGFR* activating mutation (*EGFR* deletions (ddPCR Mutation Assay *EGFR* p.L747_A750: dHsaMDV2516748; ddPCR Mutation Assay *EGFR* p.L747_S752: dHsaMDS408256419; ddPCR Mutation Assay *EGFR* E746_A750: #10041170; ddPCR *EGFR* Exon 19 Deletions Screening: #12002392), *EGFR* G719A (ddPCR Mutation Assay: dHsaMDS2512974), *EGFR* S768I (ddPCR Mutation Assay: dHsaMDV2516892) and *EGFR* L858R (ddPCR Mutation Assay: #10040783)), resistance mutations *EGFR* T790M (ddPCR Mutation Assay ID: dHsaMDV2010019), *PIK3CA* E545K (ddPCR Mutation Assay: #10041188), *PIK3CA* E542K (ddPCR Mutation Assay: #10041187) and *BRAF* V600E (ddPCR Mutation Assay: #10040779) and gene amplifications (*MET* (PrimePCR ddPCR Copy Number Assay: dHsaCP2500321), *EGFR* (PrimePCR ddPCR Copy Number Assay: dHsaCP2500318) and *ERBB2* (PrimePCR ddPCR Copy Number Assay: dHsaCP1000116)) were obtained from Bio‐Rad (Hercules, CA, USA). Additionally, primers and probes for *EGFR* exon 20 insertion (*EGFR* p.Asp770_Asn771insAlaSerValAsp) and *EGFR* C797S resistance mutations, which occur at different nucleotides within the codon for the same amino acid 797, were manually designed (*EGFR* exon 20 insertion: Primer Forward‐5′ AAATCCTCGATGAAGCCTA 3′; Primer Reverse‐5′ GGAGGTGAGGCAGATG 3′; Probe Wild Type‐HEX/BHQ‐CAGCGTGGACAACCC; Probe Insertion‐6FAM/BHQ‐CCAGCGTGGACAACC; *EGFR* C797S: Primer Forward‐5′ GCCTGCTGGGCATCTG 3′; Primer Reverse‐5′ TCTTTGTGTTCCCGGACATAGTC 3′; Probe Wild Type‐HEX/BHQ‐TTCGGCTGCCTCCTG; Probe Mutation T>A‐6FAM/BHQ‐TTCGGCAGCCTCC; Probe Mutation G>C‐6FAM/BHQ‐CTTCGGCTCCCTCCTG) and obtained from Integrated DNA Technologies (IDT, Coralville, IA, USA). Primers and probes for the detection of *ESR1* mutations (L536R, Y537C/N/S, and D538G) were designed by Schiavon et al. [39] and obtained from IDT (Coralville, IA, USA). ddPCR was performed on a QX200 Droplet Digital PCR System (Bio‐Rad, Hercules, CA, USA) according to the manufacturer's instructions, and data were analyzed using the QuantaSoft Software v.1.7.4.0917 (Bio‐Rad, Hercules, CA, USA). ctDNA quantification is presented as fractional abundance (FA—the percentage of the mutant allele in total cfDNA).

### 2.4 ctDNA Sequencing (ctDNAseq)

Targeted capture libraries were constructed from 25 ng of cfDNA using the SureSelect XT HS Target Enrichment System (Agilent Technologies, Santa Clara, CA, USA) and a panel of 151 cancer genes (ClearSeq Comprehensive Cancer Panel; Agilent Technologies, Santa Clara, CA, USA) according to the manufacturer's instructions, including modifications proposed by Mansukhani et al. [40]. Sequences were generated on a NextSeq 500 sequencing platform (Illumina, San Diego, CA, USA).

### 2.5 Variant Calling

The 150 bp paired‐end sequences were analyzed using the SureCall software (version 4.0.1.46; Agilent Technologies, Santa Clara, CA, USA) and following modifications proposed by Mansukhani et al. [40]. Variant calls were identified using the SNPPET function from SureCall, and DuplexCaller was used to identify mutations supported by duplex reads [40]. Germline variants were filtered as described elsewhere [41].

### 2.6 Statistical Analysis

This was a descriptive, real‐world cohort study. Continuous variables (e.g., patient age, time‐to‐progression, lead time between molecular and radiological progression) are summarized as medians with ranges or means, as indicated in the text; categorical variables are summarized as counts and percentages. The denominator (*n*) for each proportion is stated alongside the result (e.g., the number of evaluable patients for a given mutation or timepoint). The lead time between ctDNA‐based detection of molecular progression/resistance and RECIST‐defined radiological progression was calculated as the mean number of days between the two events for each applicable patient subgroup. All descriptive analyses and figures were generated using Microsoft Excel, GraphPad Prism (version 10.6.1, Build 799), and custom Python scripts (version 3.12.13) utilizing the Pandas (version 2.2.2) and Matplotlib (version 3.10.0) libraries.

## 3 Results

### 3.1 Patient Characteristics and Serial ctDNA Analysis

A total of 48 patients were included, comprising 42 with metastatic NSCLC and six with HR+, HER2‐negative MBC, as detailed in Tables 1 and 2, respectively. The median age of metastatic NSCLC patients was 57 years (range: 29–81). Among these, 59.5% (25/42) were female, and 71.4% (30/42) were never smokers. Thirty‐two patients were enrolled during treatment with 1st‐ and 2nd‐generation TKIs (NSCLC Cohort 1) and 10 during therapy with 3rd‐generation TKI (NSCLC Cohort 2). For the MBC cohort, the median age at initiation of endocrine therapy was 58.5 years (range: 48–71). All six patients were postmenopausal, and 83.3% (5/6) were enrolled during combined treatment of endocrine agents and iCDK4/6.

**TABLE 1:** Clinical characteristics of metastatic NSCLC patients at baseline.

Characteristics	Cohort 1 (1st/2nd‐gen EGFR TKI)	Cohort 2 (3rd‐gen EGFR TKI)	All patients
*N* (%)	32 (76.2)	10 (23.8)	42 (100)
Gender			
Female, *n* (%)	21 (65.6)	4 (40)	25 (59.5)
Male, *n* (%)	11 (34.4)	6 (60)	17 (40.5)
Median age (range)	59 (34–81)	54 (29–73)	57 (29–81)
Smoking status			
Never, *n* (%)	22 (68.8)	7 (70)	29 (69)
Ever or current smoker, *n* (%)	10 (31.2)	3 (30)	13 (31)
Stage			
IV, *n* (%)	30 (93.8)	8 (80)	38 (90.5)
Other, *n* (%)	2 (6.2)	2 (20)	4 (9.5)
Line of first TKI treatment			
1, *n* (%)	26 (81.3)	2 (20)	28 (66.6)
2, *n* (%)	5 (15.6)	5 (50)	10 (23.8)
3, *n* (%)	0 (0)	2 (20)	2 (4.8)
≥ 4, *n* (%)	1 (3.1)	1 (10)	2 (4.8)
First TKI treatment			
Erlotinib, *n* (%)	25 (78.1)		25 (59.5)
Afatinib, *n* (%)	6 (18.7)		6 (14.3)
Gefitinib, *n* (%)	1 (3.2)		1 (2.4)
Osimertinib, *n* (%)		10 (100)	10 (23.8)

**TABLE 2:** Clinical characteristics of MBC patients at baseline.

Characteristics	All patients
*N* (%)	6 (100)
Median age at endocrine therapy (ET) start (range)	58.5 (48–71)
Primary tumor histology	
Ductal, *n* (%)	4 (66.7)
Lobular, *n* (%)	2 (33.3)
Metastatic sites at ET start	
Bone only, *n* (%)	1 (16.6)
Visceral with bone, *n* (%)	5 (83.4)
Prior lines of treatment in advanced or metastatic setting	
Median (range)	3 (1–8)
No. of lines	
1, *n* (%)	2 (33.3)
2, *n* (%)	0 (0)
≥ 3, *n* (%)	4 (66.7)
ET at ESR1 mutation analysis	
AI, *n* (%)	4 (66.7)
Tamoxifen, *n* (%)	1 (16.6)
Fulvestrant, *n* (%)	1 (16.6)
CDK4/6 inhibitor	
Palbociclib, *n* (%)	4 (66.7)
Ribociclib, *n* (%)	1 (16.6)

Patient enrollment was successful overall, and monthly blood collection was performed with virtually no difficulties or complications. A variation in cfDNA yield was observed between tumor types, with lung cancer samples showing higher cfDNA concentrations than breast cancer samples. Accordingly, 3000 and 1500 genome equivalents were analyzed by ddPCR for lung and breast samples, respectively; however, this difference did not affect the accuracy or reliability of the results.

In the NSCLC cohorts, previously tumor tissue analysis for *EGFR*‐activating (*EGFR*‐act) mutations at the time of disease diagnosis showed that 71.4% (30/42) had *EGFR* exon 19 deletions, 16.7% (7/42) had *EGFR* exon 21 mutations, 4.8% (2/42) had *EGFR* exon 18 mutations, 2.4% (1/42) had *EGFR* exon 20 mutations, 2.4% (1/42) had two *EGFR*‐act mutations (*EGFR* G719S and *EGFR* S768I), and for 2.4% (1/42) information about the specific *EGFR*‐act mutation was not available. Among the 42 patients, 36 had DP and a follow‐up of 39.8 months at our institution. A total of 385 plasma samples were collected and activating and resistance mutations to EGFR TKIs were analyzed by ddPCR, averaging approximately nine samples per patient. The patient with the most collections had 48 blood samples over 5 years. Patients who benefited from 1st‐ and 2nd‐generation TKIs had an average survival of 19.9 months following metastatic diagnosis, whereas those who responded to 3rd‐generation TKIs had an average survival of 18.2 months post‐metastatic diagnosis.

For the MBC cohort, 126 plasma samples were collected, and hotspot *ESR1* mutations were analyzed using ddPCR, yielding an average of 21 samples per patient, with a maximum of 46 for one patient over 5 years and 6 months. Overall, patients had an average time to DP of 16 months after initiation of endocrine therapy and liquid biopsy monitoring.

### 3.2 Effectiveness of Liquid Biopsy for Early Detection and Characterization of Acquired Resistance to Treatment

#### 3.2.1 NSCLC Cohorts

Disease progression was observed in 29 of 32 patients (90.6%) from the NSCLC Cohort 1. Acquired resistance EGFR T790M mutation was detected in 14 out of 29 patients (48.4%) at the time of progression. *BRAF* V600E mutation was found in 1 patient (3.4%), *MET* amplification in 1 patient (3.4%), and *EGFR* amplification plus *ERBB2* amplification in 1 patient (3.4%). Additionally, transformation from NSCLC to SCLC (small cell lung cancer) was histologically confirmed in 1 patient (3.4%). We were unable to identify the molecular mechanisms of acquired resistance in 11 patients (38%) who progressed to 1st‐ and 2nd‐generation TKIs using liquid biopsy and ddPCR (Figure 1).

**FIGURE 1:**
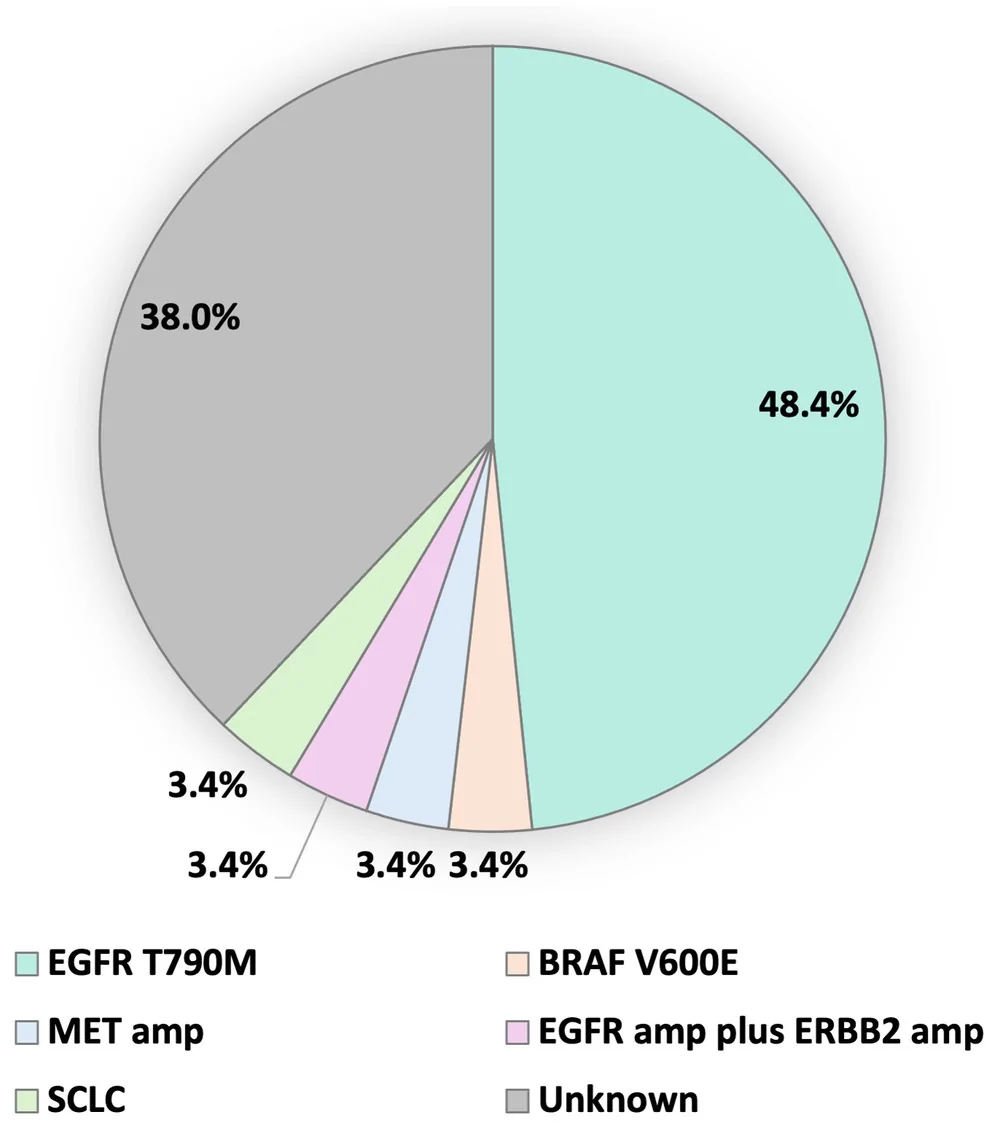
Acquired resistance mutations in metastatic NSCLC patients who have progressed to 1st‐ and 2nd‐generation TKIs. Amp, amplification; SCLC, small cell lung cancer.

Retrospective analysis was performed on serial plasma samples from 18 patients who experienced progression during treatment with 1st‐ and 2nd‐generation TKIs and were *EGFR* T790M and/or *EGFR*‐act positive at the time of DP to assess the usefulness of liquid biopsies for early detection of AR (Figure 2). The fractional abundance of *EGFR*‐act and *EGFR* T790M mutations was determined by ddPCR throughout treatment until disease progression. In 58.3% (7/12) of patients, monitoring of the *EGFR* T790M mutation revealed increases in fractional abundance that predicted progression, on average, 167 days before radiological disease progression. Similarly, in 56.2% (9/16) of patients, tracking the *EGFR*‐act mutation indicated disease progression an average of 127.5 days before radiological disease progression.

**FIGURE 2:**
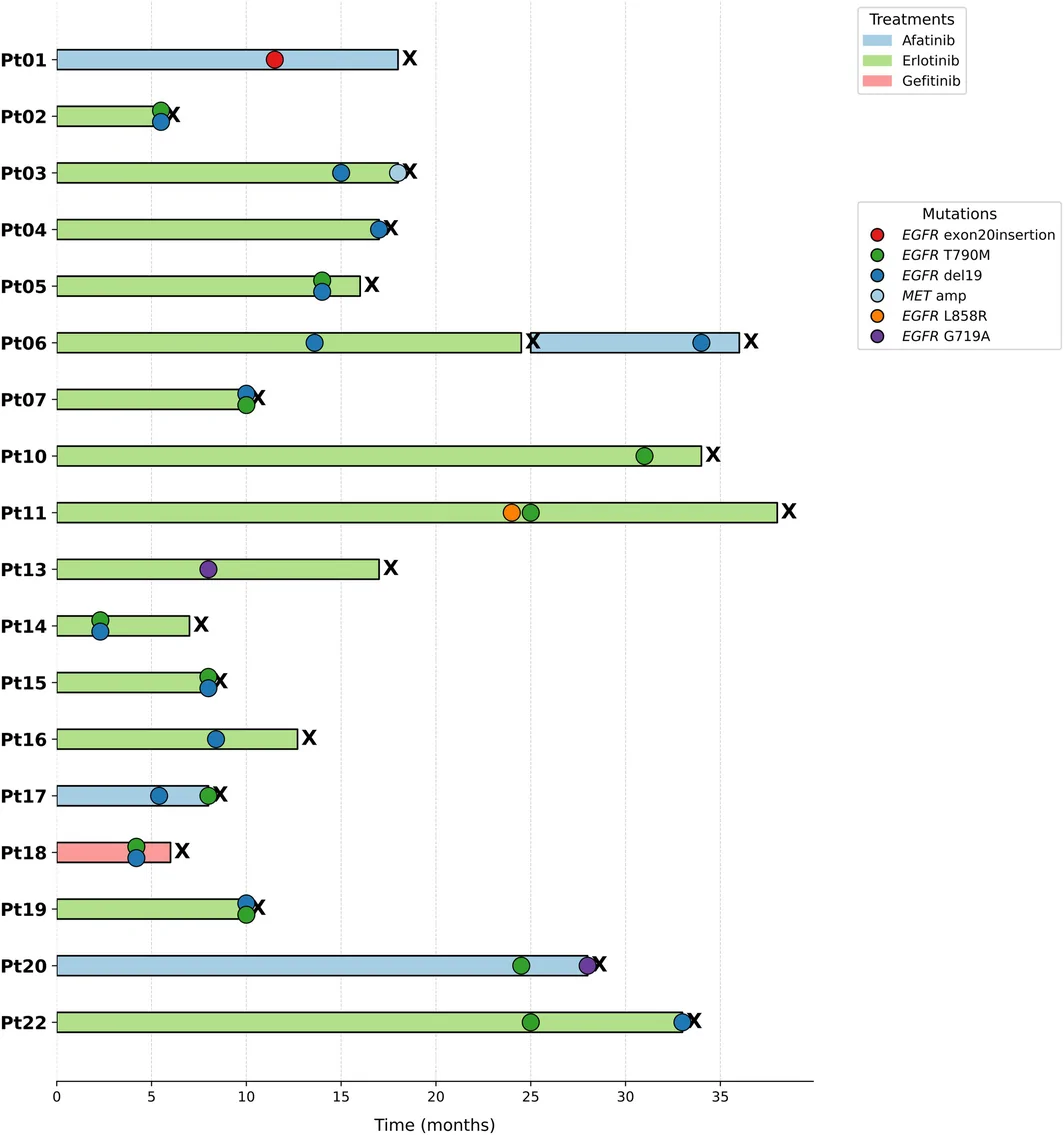
Early disease progression and resistance detection to 1st‐ and 2nd‐generation TKIs in plasma from metastatic NSCLC patients. Swimmer plot illustrating treatment duration until disease progression, indicated by an “X.” Each horizontal bar represents a patient, with segments indicating different TKI therapies. Circles denote the time points at which each mutation was detected, with mutation types distinguished by circle color as shown in the legend.

Of the 14 patients who progressed on 1st‐ and 2nd‐generation TKIs and were *EGFR* T790M‐positive, 11 (78.6%) received osimertinib and were further monitored using serial ctDNA analysis. Four additional patients, who were *EGFR* T790M‐negative and had progressed on these TKIs, also received osimertinib. In addition to these 15 patients, another 10 patients were included in our study during 3rd‐generation TKI treatment (Cohort 2). Of these 25 patients treated with 3rd‐generation TKI, 68% (17/25) experienced progression, and in 13 of them (76.5%), AR mechanisms were identified using liquid biopsies. A total of 8/17 (47%) patients were positive for *EGFR* C797S. *PIK3CA* E542K was detected in 1 patient (5.9%), *ERBB2* amplification was found in 1 patient (5.9%), and two patients harbored more than one genetic alteration associated with AR: *EGFR* C797S plus *EGFR* amplification (5.9%), and *BRAF* V600E mutation, plus *PIK3CA* E545K mutation, plus *EGFR* amplification (5.9%). Additionally, transformation to SCLC was histologically confirmed plus *EGFR* amplification (5.9%) (Figure 3).

**FIGURE 3:**
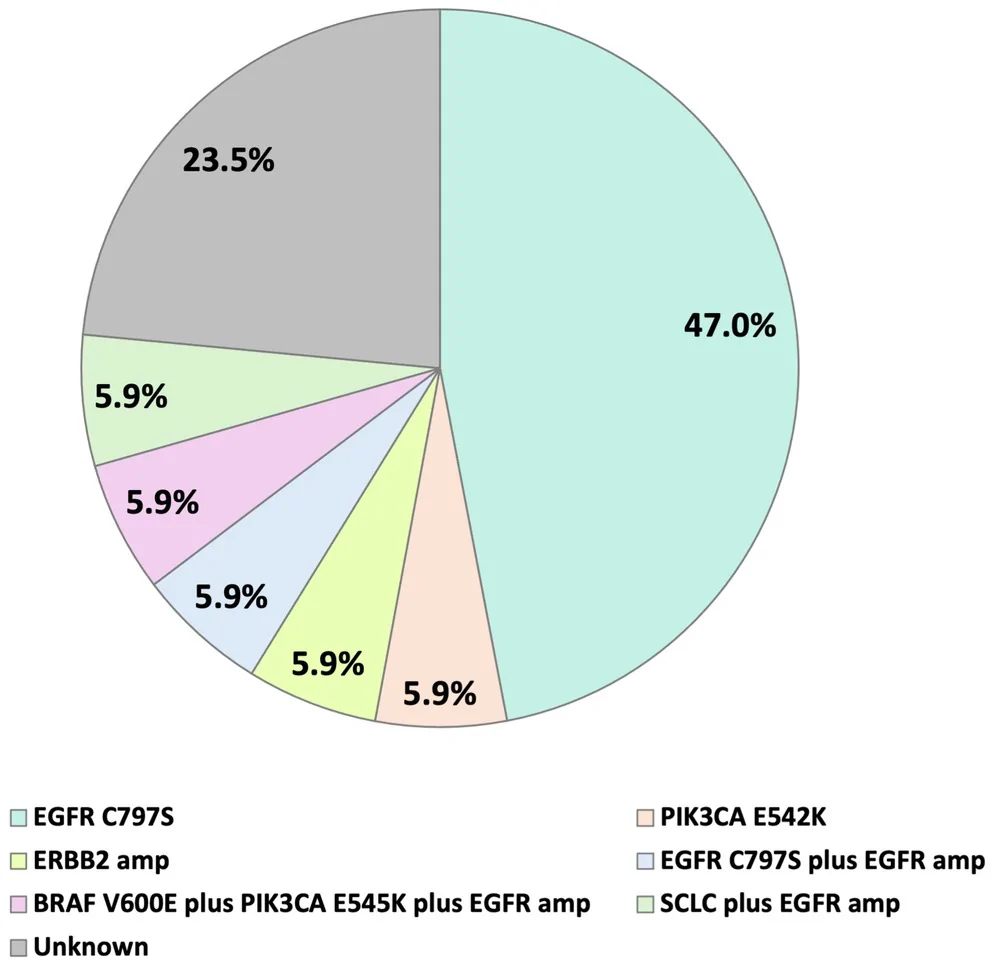
Acquired resistance mutations in metastatic NSCLC patients who progressed to 3rd‐generation TKI. Amp, amplification; SCLC, small cell lung cancer.

Serial plasma samples from 13 patients who progressed during 3rd‐generation TKI therapy and had liquid biopsies positive for AR and *EGFR*‐act mutations at the time of progression were also retrospectively analyzed to evaluate the utility of liquid biopsies for early detection of AR in patients receiving 3rd‐generation TKI (Figure 4). An increase in the fractional abundance of *EGFR*‐act predicted clinical DP by an average of 224.4 days in 92.3% (12/13) of patients, while the detection of the *EGFR*‐C797S mutated allele predicted clinical progression by an average of 62.2 days in 44.4% (4/9) patients. When considering all resistance mutations detected in all 13 patients, radiological progression was anticipated on average 145.9 days using liquid biopsies.

**FIGURE 4:**
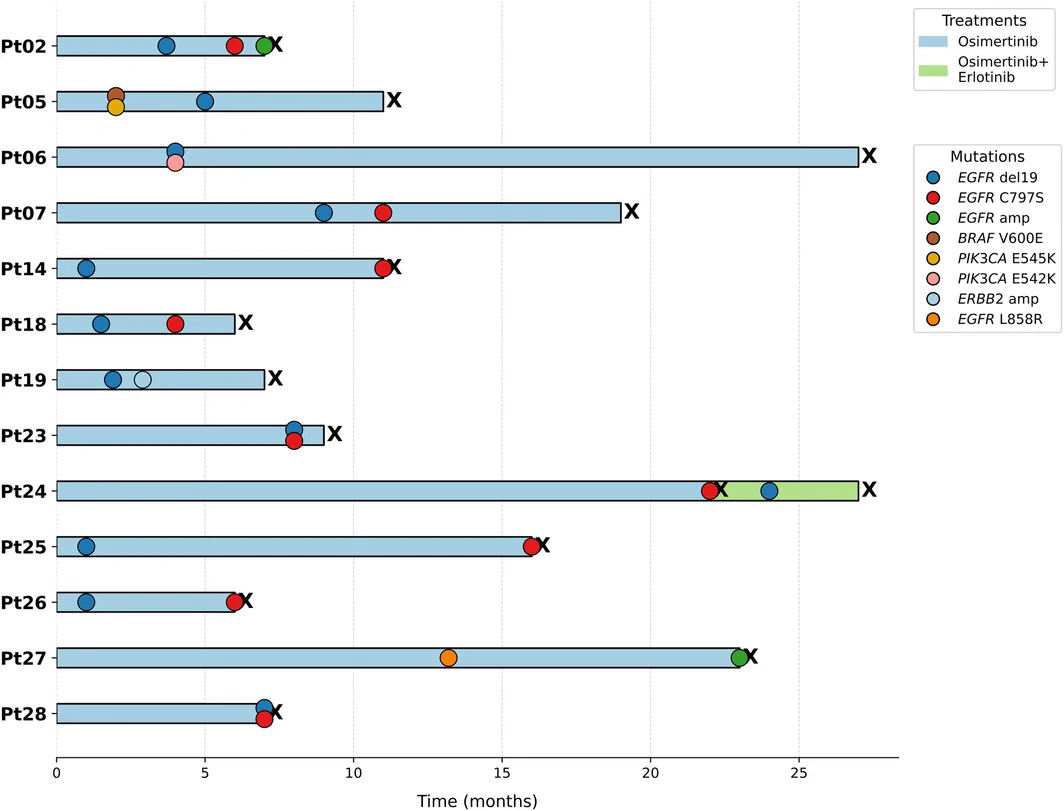
Early disease progression and resistance detection to 3rd‐generation TKI by liquid biopsies in metastatic NSCLC patients. Swimmer plot illustrating treatment duration until disease progression, indicated by an “X.” Each horizontal bar represents a patient, with segments indicating different TKI therapies. Circles denote the time points at which each mutation was detected, with mutation types distinguished by circle color as shown in the legend.

Effectiveness of liquid biopsy in molecular response monitoring was also performed and evaluated only for patients treated with 3rd‐generation TKIs, as those treated with 1st‐ and 2nd‐generation TKIs were enrolled in the study under TKI therapy. Monitoring involved detecting *EGFR*‐act and *EGFR*‐T790M mutations in 11 patients who progressed on 1st and 2nd‐generation TKIs and were positive for *EGFR*‐T790M in ctDNA. These patients received osimertinib and continued to be monitored through longitudinal plasma collections. Nine out of 11 (81.8%) patients who started osimertinib treatment had undetectable levels of the *EGFR*‐T790M mutant allele in plasma, on average 37.8 days after starting treatment, while *EGFR*‐act was negative in plasma on average 88 days after initiation of therapy, indicating a good molecular response that was later confirmed by radiological images. Monitoring of molecular response and prediction of resistance to TKI therapy through activating and resistance mutations in the ctDNA of four patients from the NSCLC cohort is illustrated in Figure 5.

**FIGURE 5:**
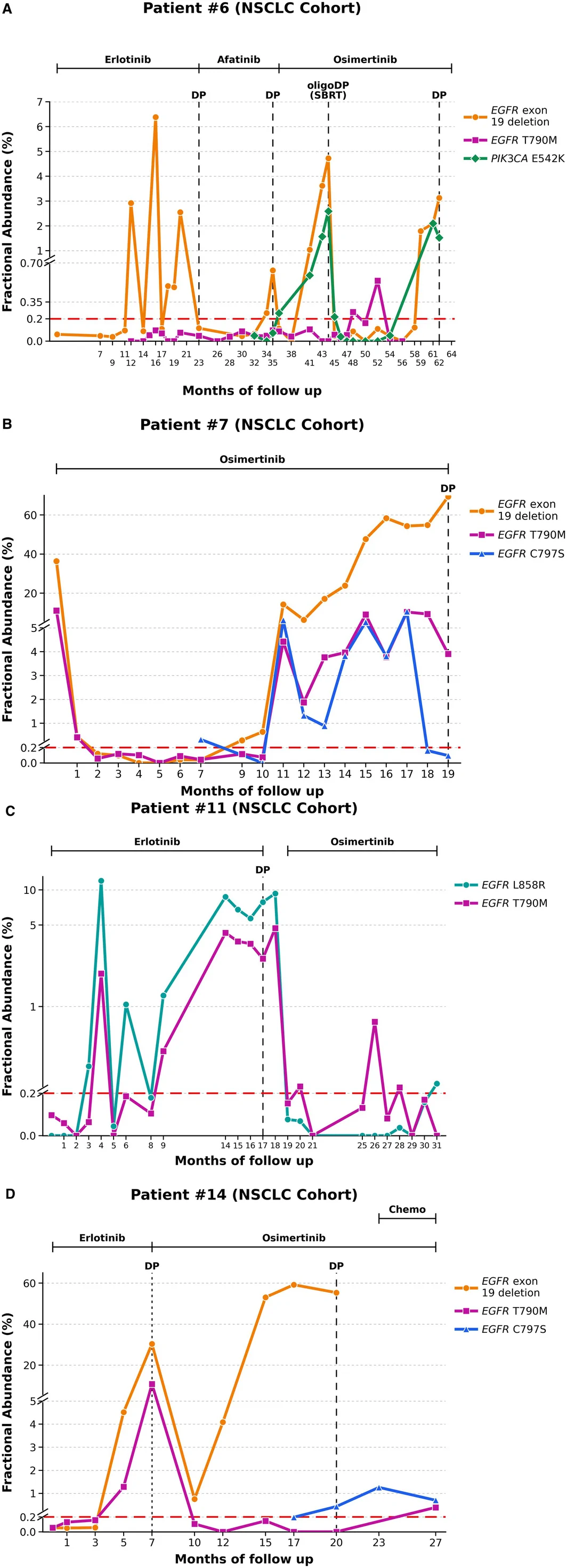
Longitudinal monitoring of EGFR‐act and resistance mutations to TKI therapy in ctDNA from patients in the NSCLC cohort. Plasma ctDNA levels are shown as the fractional abundance (FA) of each mutation detected by ddPCR. (A) Patient 6; (B) Patient 7; (C) Patient 11; (D) Patient 14. The red line indicates the limit of detection (0.2%) with FA values above this threshold considered positive for ctDNA detection. DP, disease progression. SBRT, stereotactic body radiation therapy. Chemo, chemotherapy.

#### 3.2.2 MBC Cohort

Disease progression was observed in all six patients (100%) in the MBC Cohort, and hotspot *ESR1* mutations associated with acquired resistance to ET were detected in 3 of the 6 patients (50%) (Figure 6). One patient (Pt02) had an *ESR1* D538G mutation, and another (Pt03) had an *ESR1* L536R mutation detected by ddPCR at the time of progression during treatment with ET combined with iCDK4/6. No *ESR1* mutation was detected by ddPCR at disease progression during ET plus iCDK4/6 in the third patient (Pt01). ctDNAseq was performed on the same sample, but no actionable alterations were identified. Subsequently, ctDNAseq was repeated for this patient using a sample collected at progression during fulvestrant therapy, revealing the presence of *ESR1* Y537S mutation.

**FIGURE 6:**
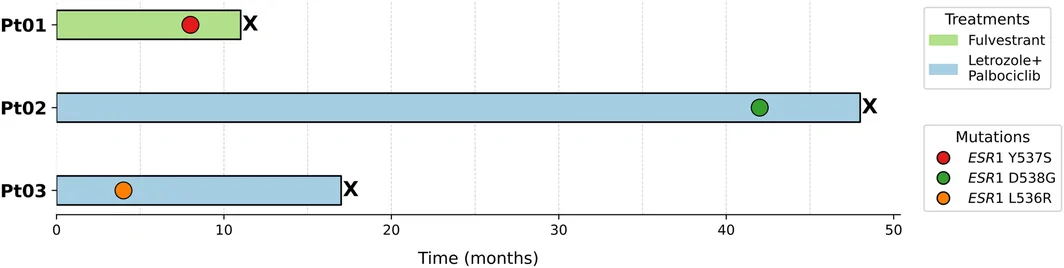
Early disease progression and resistance detection to ET in plasma from MBC patients. Swimmer plot illustrating treatment duration until disease progression, indicated by an “X.” Each horizontal bar represents a patient, with segments indicating different endocrine therapies. Circles denote the time points at which *ESR1* mutations were detected, with mutation types distinguished by circle color as shown in the legend.

The presence of these mutations was subsequently monitored by ddPCR throughout the treatment course, as shown in Figure 7. Changes in fractional abundance predicted progression, on average, 9 months before clinical progression.

**FIGURE 7:**
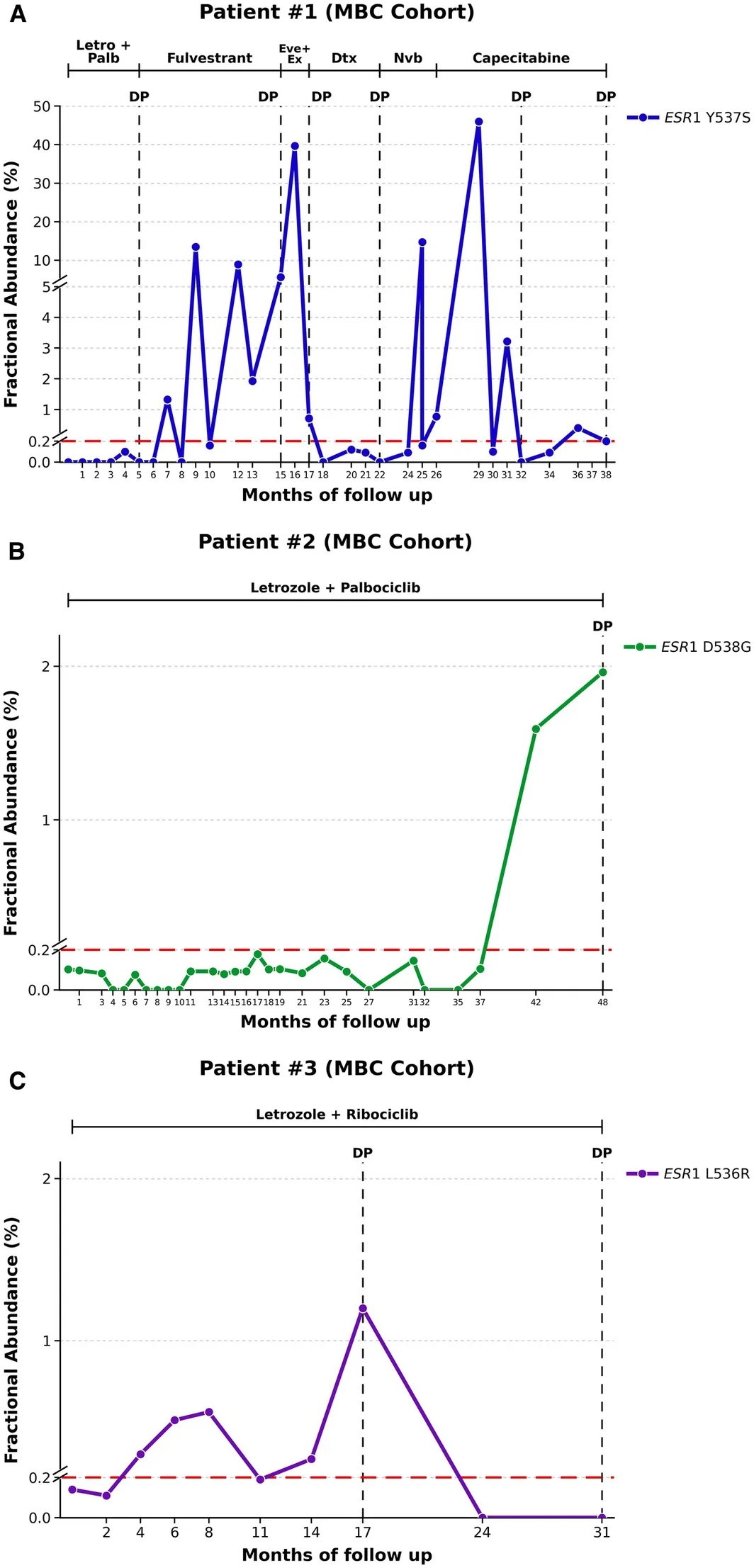
Longitudinal monitoring of *ESR1* mutations in ctDNA from patients in the MBC cohort. Plasma ctDNA levels are shown as the fractional abundance (FA) of *ESR1* mutations detected by ddPCR: (A) Patient 01—*ESR1* Y537S; (B) Patient 02—*ESR1* D538G; (C) Patient 03—*ESR1* L536R. The red line indicates the limit of detection (0.2%) with FA values above this threshold considered positive for ctDNA detection. DP, disease progression; Dtx, Docetaxel; Eve, Everolimus; Ex, Exemestane; Letro, Letrozole; Nvb, Vinorelbine; Palb, Palbociclib.

## 4 Discussion

This real‐world study demonstrates the feasibility and clinical applicability of plasma ctDNA monitoring in patients with metastatic NSCLC and HR+, HER2‐negative MBC, emphasizing the value of liquid biopsies as a minimally invasive, dynamic tool to complement standard imaging and tissue‐based methods. By repeatedly analyzing plasma samples using ddPCR, it was possible to identify molecular mechanisms of AR, predict DP before clinical progression and radiological confirmation, and assess molecular responses to targeted therapies.

In the NSCLC cohorts, our findings support previous reports that ctDNA provides clinically relevant insights into acquired resistance during TKI therapy [42, 43, 44]. *EGFR* T790M was the most common emerging resistance mutation in patients progressing on 1st/2nd‐generation TKIs (48.4%), and *EGFR* C797S predominated after 3rd‐generation TKI (47%), consistent with prior series [19, 23, 44, 45]. Longitudinal ctDNA analyses predicted DP, with increased *EGFR*‐act and *EGFR* T790M fractional abundance detected about 5 months before clinical and radiographic confirmation, underscoring ctDNA's potential as an early predictor of 1st/2nd‐generation TKI treatment failure. Additionally, after osimertinib initiation, serial ctDNA monitoring proved valuable for tracking tumor dynamics, with the quantification of *EGFR*‐act mutations providing a more sensitive and informative marker of disease burden than the detection of specific resistance alleles. As truncal mutations, *EGFR*‐act variants reflect the clonal architecture of the tumor and may therefore offer a more comprehensive view of disease evolution. It is noteworthy that, using the same approach, we previously identified amplification of an *EGFR*‐act mutation (*EGFR* exon 19 deletion) as a novel mechanism of resistance to osimertinib [38], further highlighting the versatility of ctDNA analysis in uncovering resistance pathways.

Additionally, in the osimertinib‐treated group (Cohort 2), clearance of *EGFR*‐act and *EGFR* T790M mutations shortly after therapy initiation anticipated subsequent radiological responses, confirming ctDNA monitoring as a surrogate marker of treatment effectiveness. This highlights the value of tracking both activating and resistance mutations for improved predictive accuracy.

Our results are consistent with previous studies linking ctDNA dynamics to clinical outcomes. Of note, retrospective longitudinal ctDNA analysis by ddPCR in plasma samples from the FLAURA [46] and AURA [19] phase 3 trials showed that *EGFR* mutation ctDNA monitoring could detect DP about 3 months before clinical and RECIST‐defined radiologic progression. Additionally, ctDNA clearance after 3–6 weeks of treatment was associated with improved outcomes [44].

In the MBC cohort, *ESR1* mutations associated with endocrine resistance emerged in half of patients progressing under ET plus iCDK4/6 therapy. Studies have shown that iCDK4/6 s do not prevent the appearance of *ESR1* mutations. However, these mutations do not confer resistance to iCDK4/6s [47]. Our data demonstrate that *ESR1* mutations can be tracked to monitor dynamic changes in ctDNA during therapy, offering a more precise method for detecting treatment resistance. ctDNA monitoring revealed that shifts in the fractional abundance of *ESR1* mutant alleles predicted clinical progression by nearly 9 months, aligning with previous studies that reported a median lead time of about 3–7 months [31, 48]. This observation supports the hypothesis that early detection of *ESR1* mutations could guide therapeutic decisions, as shown in the PADA‐1 trial [35], where switching therapy upon detection of emerging *ESR1* mutations improved outcomes. More recently, the phase 3 SERENA‐6 trial provided evidence supporting a ctDNA‐guided approach to detect ET resistance and adjust therapy before clinical and radiological DP in HR+, HER2‐negative advanced breast cancer [36]. Switching from ET to camizestrant, while continuing iCDK4/6 therapy based on liquid biopsy detection of *ESR1* mutations before clinical progression, resulted in improved outcomes.

It is noteworthy that the ctDNA analysis of one of the MBC patients revealed the presence of *ESR1* Y537S mutation, which is well‐established as a negative predictive biomarker of fulvestrant response [31]. Notably, this mutation was not detected at disease progression on ET plus iCDK4/6 and was only detected afterward during fulvestrant treatment. This case highlights the importance of one of the recommendations in ASCO guidelines: retesting *ESR1* wild‐type patients at subsequent progression [34]. Furthermore, a rapid disease progression was observed during treatment with exemestane (AI) plus everolimus (mTOR inhibitor), consistent with findings from the BOLERO‐2 trial. In this trial, the *ESR1* Y537S mutation was not associated with any therapeutic benefit from the combination of everolimus and an AI [49].

Taken together, our results highlight the potential of ctDNA monitoring to provide real‐time insights into tumor biology, predict DP, and guide treatment decisions in both NSCLC and HR+, HER2‐negative MBC. The minimally invasive nature of liquid biopsies enables repeated sampling, which is essential for tracking clonal dynamics and emerging resistance mechanisms that are often missed by single‐site tissue biopsies.

Nevertheless, this study has limitations. The single‐center design and relatively small number of patients in the MBC Cohort may limit its applicability. Additionally, ddPCR, while highly accurate and sensitive for predefined mutations, limits the scope of genomic profiling compared to next‐generation sequencing (NGS). In some patients, AR mechanisms could not be identified, reflecting either biological complexity or methodological limitations of using ddPCR. In contrast, for one of the MBC patients, ctDNAseq was performed, revealing an *ESR1* mutation, which complemented our analysis.

## 5 Conclusions

This real‐world study shows that ctDNA monitoring using ddPCR is a valuable tool for managing patients with metastatic NSCLC and HR+/HER2‐negative MBC. Serial plasma analysis enabled the detection of molecular mechanisms of acquired resistance, provided early prediction of disease progression compared to radiological imaging, and correlated with molecular responses to targeted therapies. These findings support integrating liquid biopsies into clinical practice as a complementary, minimally invasive, lower‐cost, and straightforward approach to tissue genotyping and imaging. Although further prospective, interventional trials are necessary to confirm the clinical benefits of ctDNA‐guided treatment decisions, our results emphasize the potential of liquid biopsies to improve personalized cancer care and optimize treatment strategies in precision oncology.

## Author Contributions

**Maurício Fernando Silva Almeida Ribeiro:** resources, formal analysis. **Franciele Hinterholz Knebel:** conceptualization, resources, methodology, investigation, formal analysis, data curation, funding acquisition, writing – original draft, writing – review and editing. **Leandro Jonata Carvalho Oliveira:** resources, writing – review and editing. **Isabela Flauzino Ferreira:** resources, methodology, investigation. **Rudinei Diogo Marques Linck:** resources. **Elisângela Monteiro Coser:** resources, methodology. **Karina Perez Sacardo:** resources. **Ernande Xavier dos Santos:** resources, methodology. **Daniele Coelho Duarte:** formal analysis, visualization, writing – review and editing. **Felipe Sales Nogueira Amorim Canedo:** resources. **Andrea Kazumi Shimada:** resources, writing – review and editing. **Paula Fontes Asprino:** methodology, investigation, writing – review and editing. **João Victor Machado Alessi:** resources. **Cibele Masotti:** formal analysis, data curation, writing – review and editing. **Max Senna Mano:** resources. **Marco Gerlinger:** formal analysis, data curation. **Frederico Perego Costa:** resources. **Fernando Costa Santini:** resources. **Artur Katz:** resources. **Dimitrios Kleftogiannis:** formal analysis, data curation. **Olavo Feher:** resources. **Louise J. Barber:** formal analysis, data curation. **Fabiana Bettoni:** conceptualization, resources, methodology, investigation, formal analysis, data curation, funding acquisition, writing – original draft, writing – review and editing. **Ciro Eduardo de Souza:** resources. **Gilberto de Castro Junior:** resources, writing – review and editing. **Anamaria Aranha Camargo:** conceptualization, formal analysis, data curation, funding acquisition, writing – original draft, writing – review and editing. **Rodrigo Saddi:** resources.

## Funding

This work was supported by grants from Sociedade Beneficente de Senhoras Hospital Sírio‐Libanês, Ludwig Institute for Cancer Research, and The São Paulo Research Foundation (FAPESP 2015/16854‐4; FAPESP 2016/05375‐0).

## Ethics Statement

The study was approved by the Hospital Sírio‐Libanês Ethics Committee (HSL2015‐22), and patients provided written informed consent for participation, clinical data collection, and genomic analysis of blood specimens.

## Consent

Patients provided written informed consent for publication of all data generated in the study.

## Conflicts of Interest

The authors declare no conflicts of interest.

## Data Availability

All data supporting the findings of this study are available from the corresponding author upon request.

## References

[1] C. Alix‐Panabieres and K. Pantel , “Liquid Biopsy: From Discovery to Clinical Application,” Cancer Discovery 11 (2021): 858–873. 10.1158/2159-8290.CD-20-1311 33811121

[2] M. Boukovala , C. B. Westphalen , and V. Probst , “Liquid Biopsy Into the Clinics: Current Evidence and Future Perspectives,” Journal of Liquid Biopsy 4 (2024): 100146. 10.1016/j.jlb.2024.100146 PMC11863819 40027149

[3] A. Bartolomucci , M. Nobrega , T. Ferrier , et al., “Circulating Tumor DNA to Monitor Treatment Response in Solid Tumors and Advance Precision Oncology,” NPJ Precision Oncology 9 (2025): 84. 10.1038/s41698-025-00876-y PMC11930993 40122951

[4] T. D. B. Russo , F. Pepe , V. Gristina , et al., “Recent Advances in Liquid Biopsy for Precision Oncology: Emerging Biomarkers and Clinical Applications in Lung Cancer,” Future Oncology 21 (2025): 2803–2821. 10.1080/14796694.2025.2542051 PMC12408062 40762271

[5] S. Loizidis , M. A. Stanciu , and M. Ignatiadis , “Liquid Biopsy in Breast Cancer: Promises and Challenges,” Current Opinion in Oncology 37 (2025): 512–521. 10.1097/CCO.0000000000001168 40657993

[6] M. Galvez‐Nino , K. Roque , R. Ruiz , et al., “Real‐World Clinical Practice and Outcomes in Peruvian Patients With Advanced EGFR T790M Mutation Positive NSCLC: A Multicenter Analysis,” Cancer Treatment and Research Communications 43 (2025): 100906. 10.1016/j.ctarc.2025.100906 40147101

[7] P. Sobczuk , K. Kozak , S. Kopec , et al., “The Use of ctDNA for BRAF Mutation Testing in Routine Clinical Practice in Patients With Advanced Melanoma,” Cancers 14 (2022): 777. 10.3390/cancers14030777 PMC8833667 35159044

[8] T. Doi , T. Ishikawa , T. Sakakida , et al., “Real‐World Genome Profiling in Japanese Patients With Pancreatic Ductal Adenocarcinoma Focusing on HRD Implications,” Cancer Science 115 (2024): 3729–3739. 10.1111/cas.16329 PMC11531956 39315592

[9] F. Canino , A. Tornincasa , S. Bettelli , et al., “Real‐World Data and Clinical Implications of Next‐Generation Sequencing (NGS)‐Based Analysis in Metastatic Breast Cancer Patients,” International Journal of Molecular Sciences 25 (2024): 2490. 10.3390/ijms25052490 PMC10931361 38473737

[10] J. Fuentes‐Antrás , A. Martínez‐Rodríguez , K. Guevara‐Hoyer , et al., “Real‐World Use of Highly Sensitive Liquid Biopsy Monitoring in Metastatic Breast Cancer Patients Treated With Endocrine Agents After Exposure to Aromatase Inhibitors,” International Journal of Molecular Sciences 24 (2023): 11419. 10.3390/ijms241411419 PMC10379453 37511178

[11] B. J. Solomon , P. J. Hesketh , L. Bazhenova , S. Shilo , and L. V. Sequist , “Clinical Relevance of Biomarker Testing in Lung Cancer,” in IASLC Atlas of Molecular Testing for Targeted Therapy in Lung Cancer, vol. 2, 1st ed., ed. L. M. Sholl , W. A. Cooper , K. M. Kerr , D. S. W. Tan , M.‐S. Tsao , and J. C.‐H. Yang (International Association for the Study of Lung Cancer; Chapter, 2023), 7–19.

[12] A. Midha , S. Dearden , and R. McCormack , “EGFR Mutation Incidence in Non‐Small‐Cell Lung Cancer of Adenocarcinoma Histology: A Systematic Review and Global Map by Ethnicity (mutMapII),” American Journal of Cancer Research 5 (2015): 2892–2911. PMC4633915 26609494

[13] L. V. Sequist , J. C. Yang , N. Yamamoto , et al., “Phase III Study of Afatinib or Cisplatin Plus Pemetrexed in Patients With Metastatic Lung Adenocarcinoma With EGFR Mutations,” Journal of Clinical Oncology 31 (2013): 3327–3334. 10.1200/JCO.2012.44.2806 23816960

[14] R. Rosell , E. Carcereny , R. Gervais , et al., “Erlotinib Versus Standard Chemotherapy as First‐Line Treatment for European Patients With Advanced EGFR Mutation‐Positive Non‐Small‐Cell Lung Cancer (EURTAC): A Multicentre, Open‐Label, Randomised Phase 3 Trial,” Lancet Oncology 13 (2012): 239–246. 10.1016/S1470-2045(11)70393-X 22285168

[15] S. S. Ramalingam , J. Vansteenkiste , D. Planchard , et al., “Overall Survival With Osimertinib in Untreated, EGFR‐ Mutated Advanced NSCLC,” New England Journal of Medicine 382 (2020): 41–50. 10.1056/NEJMoa1913662 31751012

[16] L. Paz‐Ares , E.‐H. Tan , K. O'Byrne , et al., “Afatinib Versus Gefitinib in Patients With EGFR Mutation‐Positive Advanced Non‐Small‐Cell Lung Cancer: Overall Survival Data From the Phase IIb LUX‐Lung 7 Trial,” Annals of Oncology 28 (2017): 270–277. 10.1093/annonc/mdw611 PMC5391700 28426106

[17] A. Inoue , K. Kobayashi , M. Maemondo , et al., “Updated Overall Survival Results From a Randomized Phase III Trial Comparing Gefitinib With Carboplatin‐Paclitaxel for Chemo‐Naive Non‐Small Cell Lung Cancer With Sensitive EGFR Gene Mutations (NEJ002),” Annals of Oncology 24 (2013): 54–59. 10.1093/annonc/mds214 22967997

[18] M. Schuler , Y. L. Wu , V. Hirsh , et al., “First‐Line Afatinib Versus Chemotherapy in Patients With Non‐Small Cell Lung Cancer and Common Epidermal Growth Factor Receptor Gene Mutations and Brain Metastases,” Journal of Thoracic Oncology 11 (2016): 380–390. 10.1016/j.jtho.2015.11.014 26823294

[19] T. S. Mok , Y.‐L. Wu , M.‐J. Ahn , et al., “Osimertinib or Platinum‐Pemetrexed in EGFR T790M‐Positive Lung Cancer,” New England Journal of Medicine 376 (2017): 629–640. 10.1056/NEJMoa1612674 PMC6762027 27959700

[20] G. Recondo , F. Facchinetti , K. A. Olaussen , B. Besse , and L. Friboulet , “Making the First Move in EGFR‐Driven or ALK‐Driven NSCLC: First‐Generation or Next‐ Generation TKI?,” Nature Reviews Clinical Oncology 15 (2018): 694–708. 10.1038/s41571-018-0081-4 30108370

[21] C. S. Tan , D. Gilligan , and S. Pacey , “Treatment Approaches for EGFR‐Inhibitor‐ Resistant Patients With Non‐Small‐Cell Lung Cancer,” Lancet Oncology 16 (2015): e447–e459. 10.1016/S1470-2045(15)00246-6 26370354

[22] L. V. Sequist , B. A. Waltman , D. Dias‐Santagata , et al., “Genotypic and Histological Evolution of Lung Cancers Acquiring Resistance to EGFR Inhibitors,” Science Translational Medicine 3 (2011): 75ra26. 10.1126/scitranslmed.3002003 PMC3132801 21430269

[23] D. R. Camidge , W. Pao , and L. V. Sequist , “Acquired Resistance to TKIs in Solid Tumours: Learning From Lung Cancer,” Nature Reviews. Clinical Oncology 11 (2014): 473–481. 10.1038/nrclinonc.2014.104 24981256

[24] J. F. Gainor and A. T. Shaw , “Emerging Paradigms in the Development of Resistance to Tyrosine Kinase Inhibitors in Lung Cancer,” Journal of Clinical Oncology 31 (2013): 3987–3996. 10.1200/JCO.2012.45.2029 PMC3805932 24101047

[25] A. Leonetti , S. Sharma , R. Minari , P. Perego , E. Giovannetti , and M. Tiseo , “Resistance Mechanisms to Osimertinib in EGFR‐Mutated Non‐Small Cell Lung Cancer,” British Journal of Cancer 121 (2019): 725–737. 10.1038/s41416-019-0573-8 PMC6889286 31564718

[26] A. K. Güren , E. Kocaaslan , Y. Ağyol , et al., “Acquired *CD74‐ROS1* Fusion‐Mediated Osimertinib Resistance Successfully Treated With Osimertinib and Crizotinib: A Case Report,” Journal of Chemotherapy (2026): 1–5, 10.1080/1120009X.2026.2657116. 41992733

[27] C. Rolfo , P. Mack , G. V. Scagliotti , et al., “Liquid Biopsy for Advanced NSCLC: A Consensus Statement From the International Association for the Study of Lung Cancer,” Journal of Thoracic Oncology 16 (2021): 1647–1662. 10.1016/j.jtho.2021.06.017 34246791

[28] A. Gennari , F. André , C. H. Barrios , et al., “ESMO Clinical Practice Guideline for the Diagnosis, Staging and Treatment of Patients With Metastatic Breast Cancer,” Annals of Oncology 32 (2021): 1475–1495. 10.1016/j.annonc.2021.09.019 34678411

[29] H. J. Burstein , M. R. Somerfield , D. L. Barton , et al., “Endocrine Treatment and Targeted Therapy for Hormone Receptor‐Positive, Human Epidermal Growth Factor Receptor 2‐Negative Metastatic Breast Cancer: ASCO Guideline Update,” Journal of Clinical Oncology 39 (2021): 3959–3977. 10.1200/JCO.21.01392 PMC8659999 34324367

[30] A. K. Güren , M. F. Kircali , O. Köstek , and N. C. Demircan , “Post‐CDK4/6 Inhibitor Treatment Landscape in Metastatic Hormone Receptor‐Positive Breast Cancer: A Narrative Review,” Expert Review of Anticancer Therapy 26 (2025): 425–435. 10.1080/14737140.2025.2593540 41267635

[31] J. O. Brett , L. M. Spring , A. Bardia , and S. A. Wander , “ESR1 Mutation as an Emerging Clinical Biomarker in Metastatic Hormone Receptor‐Positive Breast Cancer,” Breast Cancer Research 23 (2021): 85. 10.1186/s13058-021-01462-3 PMC8365900 34392831

[32] F. C. Bidard , V. G. Kaklamani , P. Neven , et al., “Elacestrant (Oral Selective Estrogen Receptor Degrader) Versus Standard Endocrine Therapy for Estrogen Receptor‐Positive, Human Epidermal Growth Factor Receptor 2‐Negative Advanced Breast Cancer: Results From the Randomized Phase III EMERALD Trial,” Journal of Clinical Oncology 40 (2022): 3246–3256. 10.1200/JCO.22.00338 PMC9553388 35584336

[33] N. Turner , C. Huang‐Bartlett , K. Kalinsky , et al., “Design of SERENA‐6, a Phase III Switching Trial of Camizestrant in ESR1‐Mutant Breast Cancer During First‐Line Treatment,” Future Oncology 19 (2023): 559–573. 10.2217/fon-2022-1196 37070653

[34] H. J. Burstein , A. DeMichele , M. R. Somerfield , N. L. Henry , and Biomarker Testing and Endocrine and Targeted Therapy in Metastatic Breast Cancer Expert Panels , “Testing for ESR1 Mutations to Guide Therapy for Hormone Receptor‐Positive, Human Epidermal Growth Factor Receptor 2‐Negative Metastatic Breast Cancer: ASCO Guideline Rapid Recommendation Update,” Journal of Clinical Oncology 41 (2023): 3423–3425. 10.1200/JCO.23.00638 37196213

[35] F. C. Bidard , A. C. Hardy‐Bessard , F. Dalenc , et al., “Switch to Fulvestrant and Palbociclib Versus no Switch in Advanced Breast Cancer With Rising ESR1 Mutation During Aromatase Inhibitor and Palbociclib Therapy (PADA‐1): A Randomised, Open‐Label, Multicentre, Phase 3 Trial,” Lancet Oncology 23 (2022): 1367–1377. 10.1016/S1470-2045(22)00555-1 36183733

[36] F. C. Bidard , E. L. Mayer , Y. H. Park , et al., “First‐Line Camizestrant for Emerging ESR1‐Mutated Advanced Breast Cancer,” New England Journal of Medicine 393 (2025): 569–580. 10.1056/NEJMoa2502929 40454637

[37] E. A. Eisenhauer , P. Therasse , J. Bogaerts , et al., “New Response Evaluation Criteria in Solid Tumours: Revised RECIST Guideline (Version 1.1),” European Journal of Cancer 45 (2009): 228–247. 10.1016/j.ejca.2008.10.026 19097774

[38] F. H. Knebel , F. Bettoni , A. K. Shimada , et al., “Sequential Liquid Biopsies Reveal Dynamic Alterations of EGFR Driver Mutations and Indicate EGFR Amplification as a New Mechanism of Resistance to Osimertinib in NSCLC,” Lung Cancer 108 (2017): 238–241. 10.1016/j.lungcan.2017.04.004 28625643

[39] G. Schiavon , S. Hrebien , I. Garcia‐Murillas , et al., “Analysis of ESR1 Mutation in Circulating Tumor DNA Demonstrates Evolution During Therapy for Metastatic Breast Cancer,” Science Translational Medicine 7 (2015): 313ra182. 10.1126/scitranslmed.aac7551 PMC4998737 26560360

[40] S. Mansukhani , L. J. Barber , D. Kleftogiannis , et al., “Ultra‐Sensitive Mutation Detection and Genome‐Wide DNA Copy Number Reconstruction by Error Corrected Circulating Tumour DNA Sequencing,” Clinical Chemistry 64 (2018): 1626–1635. 10.1373/clinchem.2018.289629 PMC6214522 30150316

[41] M. M. Queiroz , R. R. Munhoz , C. Masotti , et al., “Durable Response to Nivolumab in a Patient With Hepatic Sarcomatoid Carcinoma: Evolutive Characterization of Genomic and Immunohistochemical PD‐L1 Expression Findings,” JCO Precision Oncology 6 (2022): e2200163. 10.1200/PO.22.00163 PMC9489170 36044716

[42] C. T. Huang , C. A. Lin , T. J. Su , et al., “Monitoring of T790M in Plasma ctDNA of Advanced EGFR‐Mutant NSCLC Patients on First‐ or Second‐Generation Tyrosine Kinase Inhibitors,” BMC Cancer 23 (2023): 234. 10.1186/s12885-023-10698-5 PMC10010021 36915101

[43] B. S. Sorensen , L. Wu , W. Wei , et al., “Monitoring of Epidermal Growth Factor Receptor Tyrosine Kinase Inhibitor‐Sensitizing and Resistance Mutations in the Plasma DNA of Patients With Advanced Non‐Small Cell Lung Cancer During Treatment With Erlotinib,” Cancer 120 (2014): 3896–3901. 10.1002/cncr.28964 PMC4303984 25103305

[44] J. E. Gray , A. Markovets , T. Reungwetwattana , et al., “Longitudinal Analyses of Circulating Tumor DNA for the Detection of EGFR Mutation‐Positive Advanced NSCLC Progression During Treatment: Data From FLAURA and AURA3,” Journal of Thoracic Oncology 19 (2024): 1525–1538. 10.1016/j.jtho.2024.07.008 39029876

[45] X. Le , S. Puri , M. V. Negrão , et al., “Landscape of EGFR‐Dependent and ‐Independent Resistance Mechanisms to Osimertinib and Continuation Therapy Beyond Progression in EGFR‐Mutant NSCLC,” Clinical Cancer Research 24 (2018): 6195–6203. 10.1158/1078-0432.CCR-18-1542 PMC6295279 30228210

[46] J. C. Soria , Y. Ohe , J. Vansteenkiste , et al., “Osimertinib in Untreated EGFR‐Mutated Advanced Non–Small‐Cell Lung Cancer,” New England Journal of Medicine 378 (2018): 113–125. 10.1056/NEJMoa1713137 29151359

[47] B. O'Leary , R. J. Cutts , Y. Liu , et al., “The Genetic Landscape and Clonal Evolution of Breast Cancer Resistance to Palbociclib Plus Fulvestrant in the PALOMA‐3 Trial,” Cancer Discovery 8 (2018): 1390–1403. 10.1158/2159-8290.CD-18-0264 PMC6368247 30206110

[48] C. Fribbens , I. Garcia‐Murillas , M. Beaney , et al., “Tracking Evolution of Aromatase Inhibitor Resistance With Circulating Tumour DNA Analysis in Metastatic Breast Cancer,” Annals of Oncology 29 (2018): 145–153. 10.1093/annonc/mdx483 PMC6264798 29045530

[49] S. Chandarlapaty , D. Chen , W. He , et al., “Prevalence of ESR1 Mutations in Cell‐Free DNA and Outcomes in Metastatic Breast Cancer: A Secondary Analysis of the BOLERO‐2 Clinical Trial,” JAMA Oncology 2 (2016): 1310–1315. 10.1001/jamaoncol.2016.1279 PMC5063698 27532364

